# Polybenzimidazole Confined in Semi-Interpenetrating Networks of Crosslinked Poly (Arylene Ether Ketone) for High Temperature Proton Exchange Membrane

**DOI:** 10.3390/nano12050773

**Published:** 2022-02-25

**Authors:** Erli Qu, Junqiao Jiang, Min Xiao, Dongmei Han, Sheng Huang, Zhiheng Huang, Shuanjin Wang, Yuezhong Meng

**Affiliations:** 1 The Key Laboratory of Low-Carbon Chemistry & Energy Conservation of Guangdong Province, State Key Laboratory of Optoelectronic Materials and Technologies, School of Materials Science and Engineering, Sun Yat-sen University, Guangzhou 510275, China; querli@mail2.sysu.edu.cn (E.Q.); jiangjq5@mail2.sysu.edu.cn (J.J.); stsxm@mail.sysu.edu.cn (M.X.); handongm@mail.sysu.edu.cn (D.H.); huangsh47@mail.sysu.edu.cn (S.H.); hzh29@mail.sysu.edu.cn (Z.H.); 2 School of Chemical Engineering and Technology, Sun Yat-Sen University, Zhuhai 519082, China

**Keywords:** high-temperature proton exchange membrane, semi-interpenetration network, phosphoric acid, poly [2,2′-(p-oxdiphenylene)-5,5′-benzimidazole]

## Abstract

As a traditional high-temperature proton exchange membrane (HT-PEM), phosphoric acid (PA)-doped polybenzimidazole (PBI) is often subject to severe mechanical strength deterioration owing to the “plasticizing effect” of a large amount of PA. In order to address this issue, we fabricated the HT-PEMs with a crosslinked network of poly (arylene ether ketone) to confine polybenzimidazole in semi-interpenetration network using self-synthesized amino-terminated PBI (PBI-4NH_2_) as a crosslinker. Compared with the pristine linear poly [2,2′-(p-oxdiphenylene)-5,5′-benzimidazole] (OPBI) membrane, the designed HT-PEMs (semi-IPN/*x*PBI), in the semi-IPN means that the membranes with a semi-interpenetration structure and *x* represent the combined weight percentage of PBI-4NH_2_ and OPBI. In addition, they also demonstrate an enhanced anti-oxidative stability and superior mechanical properties without the sacrifice of conductivity. The semi-IPN/70PBI exhibits a higher proton conductivity than OPBI at temperatures ranging from 80 to 180 °C. The HT-PEMFC with semi-IPN/70PBI exhibits excellent H_2_/O_2_ single cell performance with a power density of 660 mW cm^−2^ at 160 °C with flow rates of 250 and 500 mL min^−1^ for dry H_2_ and O_2_ at a backpressure of 0.03 MPa, which is 18% higher than that of OPBI (561 mW cm^−2^) under the same test conditions. The results indicate that the introduction of PBI containing crosslinked networks is a promising approach to improve the comprehensive performance of HT-PEMs.

## 1. Introduction

In the last few decades, high temperature proton exchange membrane fuel cells (HT-PEMFCs) with an operating temperature above 100 °C and a relative humidity below 50% have drawn increasing attention as advanced energy conversion devices, owing to their remarkable advantages of excellent thermal efficiency, high catalyst tolerance to CO, rapid electrode kinetics, simple water-heat management systems, and so on [1,2,3,4,5]. As well established, the proton exchange membranes (PEMs) as the core part of HT-PEMFCs have an important impact on the lifespan and performance of fuel cells, which serves to transport protons and avoid fuel penetration from two electrodes [6,7,8]. Accordingly, HT-PEMs were extensively studied by many researchers in order to meet the advanced HT-PEMFC technology [9,10]. To date, phosphoric acid-doped polybenzimidazole (PA-PBI) is rapidly becoming one of the most promising candidates to dominate the HT-PEMs prospect due to its favorable proton conductivity and its outstanding thermal and chemical stability without humidification conditions at elevated temperatures [11,12,13,14]. PA-PBI membrane has been widely investigated and developed since it was first reported by Wainright et al. in 1995 [15]. For PA-PBI membrane, the proton conductivity increases with increasing acid doping levels (ADLs). In far too many circumstances, a high PA doping level is essential to obtain the ideal proton conductivity for PA-PBI-based HT-PEMFCs. Nevertheless, excessive ADLs of the membranes result in the deterioration of mechanical strength, dimensional stability, and chemical stability on account of the “plasticizing effect” of the doped acid and the dissolution of PBI [16,17,18,19]. Moreover, excessive PA is apt to leach out in the period of cell operation due to the condensation of water in the cathode, which in turn worsens the cell performance [20,21,22]. The above two key aspects restrict the practical application of PBI-based membranes for PEMs.

In order to address the trade-off between proton conductivity and mechanical strength of PA doped PBI-based membranes, many attempts have been made, such as preparing cross-linked membranes [23], copolymerization membranes [24], acid-base blend membranes [25], and hybrid membranes with inorganic fillers [26]. Among these methods, cross-linking has been proven to be a significant approach to strengthen the comprehensive performance of PA-PBI membranes as HT-PEMs, notably in the respect of oxidative stability, acid retention ability, anti-degradation, and mechanical strength/dimensional stability [27,28,29,30,31,32], because it can connect chemical bonds and polymer molecules by chemical bonds to form a network or body structure. Over the past decades, the research on cross-linked PBI has been a concern of researchers. However, there are two issues that need to be taken seriously. On the one hand, the cross-linked membranes with a high crosslinking degree will be brittle and difficult to prepare. On the other hand, the PA absorption of the cross-linked membranes will be restricted because cross-linking leads to a compact molecular chain in the membranes [20,33]. For instance, Li et al. reported that the ADL of the as-prepared cross-linked PBI membranes via p-xylene dibromide cross-linking agent reduced from 15.5 to 8.5 PA per unit, compared with the linear PBI membrane [34]. Kim et al. prepared a crosslinked P(pBUa-co-BI)-20 membrane, however, its conductivity was 30% lower than that of original PBI [35]. Moreover, the conventional cross-linker is utilized at the expense of the concentration of active N-H groups, and with the amount of crosslinking agents increasing, a more compact product will be produced [36,37]. These negative factors will inevitably reduce ADLs, and consequently, decrease the proton conductivity and weaken the single-cell performance [38,39,40]. For example, compared with pure PBI, the ADL of a crosslinked PBI membrane dropped from 13.9 to 10.3, thus leading to a sharp decrease in conductivity from 144 mS cm^−1^ to 66 mS cm^−1^ at 180 °C [30]. Therefore, to obtain higher proton conductivity under lower acid uptake and to enhance fuel cell performance, it is crucial to design and develop a new crosslinking agent without the elimination of N-H group on the imidazole ring.

Poly (arylene ether ketone) (PAEK) in particular is well known as an ideal PEM supplementary material, because of its excellent mechanical strength, favorable thermal stability, outstanding solubility, and suitability for mass production [41,42,43,44]. Furthermore, poly [2,2′-(p-oxdiphenylene)-5,5′-benzimidazole] (OPBI) has been widely studied because it contains ether bonds, which are conducive to the absorption of PA and proton conduction.

In this work, we prepared HT-PEMs (semi-IPN/*x*PBI) with a semi-interpenetration structure, in which the linear OPBI is confined in the crosslinked network of PAEK by a designed PBI-based crosslinker (PBI-4NH_2_). A kind of neoteric crosslinking agent PBI-4NH_2_ with amino ended-groups was designed and synthesized, which can provide active imidazole groups in the crosslinked network. Compared to the pristine linear OPBI, the resultant semi-IPN/*x*PBI exhibited superior physicochemical stabilities. Upon doping PA, the composite membrane presents an enhanced electrochemical performance. 

## 2. Experimental

### 2.1. Materials

Poly [2,2′-(p-oxdiphenylene)-5,5′-benzimidazole] (OPBI, 6000 Pa·S) was purchased from Shanghai Shengjun Plastic Technology Co., Ltd. (Shanghai, China). Phenolphthalin (PPL), 3,3′-Diaminobenzidine (DAB, 97%), isophthalic acid (IPA), and 4,4′-difluorobenzophenone (99%) were acquired from Shanghai Macklin Biochemical Co. Ltd. (Shanghai, China). Anhydrous potassium carbonate (K_2_CO_3_), hydrochloric acid (HCl), and toluene were obtained from Guangzhou Chemical Reagent Factory (Guangzhou, China). Phosphoric acid solution (85 wt.%), phosphorus pentoxide (P_2_O_5_), polyphosphoric acid (PPA), N, N-Dimethylacetamide (DMAc), and 1-Methyl-2-pyrrolidinone (NMP) were purchased from Aladdin Chemistry Co. Ltd. (Shanghai, China). Ammonia solution (25 wt.%) was acquired from Tianjin Fuyu Fine Chemical Co. Ltd. (Tianjin, China). All the materials were acquired commercially and utilized as received.

### 2.2. Methods

#### 2.2.1. Preparation of Poly (Arylene Ether Ketone) with Pendant Carboxyl Groups (PAEK-COOH)

**Scheme 1:**
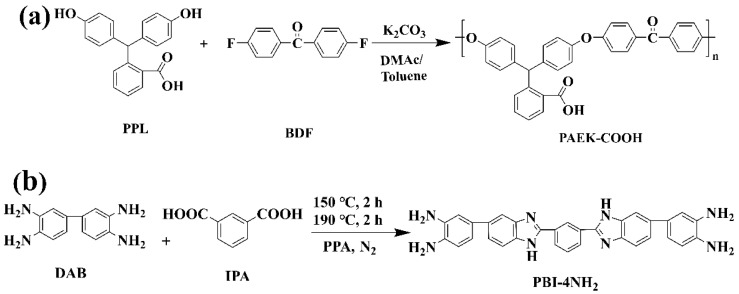
Synthesis of (**a**) PAEK-COOH, and (**b**) PBI-4NH_2_.

As depicted in Scheme 1a, PAEK-COOH was prepared via a typical nucleophilic aromatic substitution reaction. The detailed polycondensation reaction presented here was performed as follows: 4,4′-difluorobenzophenone (2.18 g, 10 mmol), PPL (3.20 g, 10 mmol), and K_2_CO_3_ (3.46 g, 30 mmol) were added to a 150 mL three-neck round-bottomed flask furnished with a Dean—Stark trap containing a reflux condenser, a magnetic stirrer, and a nitrogen inlet. In the meanwhile, DMAc and toluene were added as the solvent and water removal system to form a solid concentration of 21 wt.%. The reaction mixture was firstly stirred and maintained at the reflux temperature of about 140 °C for 4–5 h so that the water was completely removed from the reaction system, and then the toluene was removed. Next, the reaction temperature was slowly promoted to 170 °C and maintained for about 24 h until the polymerization was completed, during which the reaction solution became more viscous. After cooling to room temperature, the reacted viscous solution was precipitated in the mixed solution of concentrated HCl, CH_3_OH, and deionized water and stirred vigorously. The resulting threadlike polymer was further crushed into powder through a high-speed blender and filtered. Finally, the light green powder was washed several times sequentially using deionized water and ethanol, respectively, and dried in a vacuum oven at 100 °C for 24 h.

#### 2.2.2. Preparation of Amino-Ended Crosslinker (PBI-4NH_2_)

As shown in Scheme 1b, a crosslinker of PBI-4NH_2_ was prepared by means of a condensation reaction between isophthalic acid (IPA) and 3,3′-diaminobenzidine (DAB) using PPA as a catalyst and dehydrating agent. The specific implementation process is as follows: firstly, 81 g of PPA and 21 g of P_2_O_5_ were put into a 150 mL three-neck round-bottomed flask equipped with a mechanical stirrer and a nitrogen inlet and outlet. Then, the mixture was stirred at 120 °C for approximately 4 h until the solution was transparent. Secondly, the solution was cooled to room temperature, and DAB (3.8569 g, 18 mmol) was added to the flask and stirred at 150 °C to dissolve it completely. Next, IPA (1.4952 g, 9 mmol) was added in batches and stirred for 2 h at 150 °C. Finally, the reaction temperature was increased to 190 °C and kept for another 22 h. After the condensation reaction, the viscous solution was slowly poured into ice deionized water. In order to neutralize the excess phosphoric acid, a dilute solution of ammonium hydroxide (10 wt.%) was used to adjust the pH to neutral. In the last step, the resulting oligomer was filtered, washed with distilled water several times, and dried using a freeze dryer at −60 °C for 48 h so that the residual solvent was completely removed.

#### 2.2.3. Preparation of Semi-IPN/*x*PBI Membranes

**Scheme 2:**
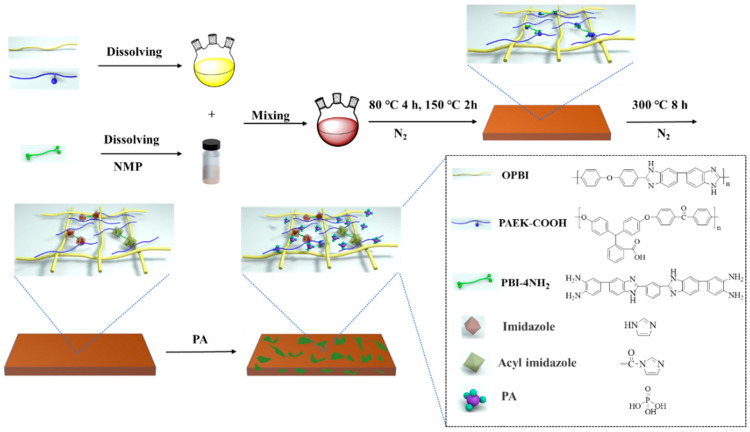
Preparation of semi-IPN/*x*PBI membranes.

As depicted in Scheme 2, the designed membranes with a semi-IPN structure were prepared by the solution casting method with an in situ crosslinking reaction between PAEK-COOH and PBI-4NH_2_. The obtained membranes are named semi-IPN/*x*PBI, in which semi-IPN means the membranes with a semi-interpenetration structure, and *x* represents the combined weight percentage of PBI-4NH_2_ and OPBI. For an easier comparison, the pure OPBI membrane and the fully crosslinked membrane of PAEK-COOH and PBI-4NH_2_ (denoted as PAEK-*cr*-PBI) were also prepared separately. The details are depicted as follows.

OPBI membrane: 1.0 g OPBI powder was dissolved in 10 mL NMP solvent under continuous magnetic stirring at 80 °C for 12 h to form a transparent claybank polymer solution, and then naturally cooled to room temperature. The solution was cast on a clean glass plate and then treated at 80 °C for 12 h to obtain OPBI membrane.

PAEK-*cr*-PBI membrane: the molar ratio of the carboxyl groups of PAEK-COOH to the amino groups of PBI-4NH_2_ was 1:2. PAEK-COOH (746.3 mg) and PBI-4NH_2_ (392.0 mg) were dissolved into 5 mL and 3 mL NMP, respectively. Subsequently, the solution of PBI-4NH_2_ was added into PAEK-COOH solution drop by drop under vigorous magnetic stirring. After that, the mixed solution was stirred at 80 °C for 4 h and 150 °C for 2 h under N_2_ atmosphere to complete the pre-crosslinking process. After that, the pre-crosslinking solution was cooled to about 60 °C and cast onto a clean glass plate and heated at 80 °C for 12 h to volatilize the solvent to obtain the pre-crosslinked membrane. Next, the as-prepared pre-crosslinked membrane was further treated under N_2_ atmosphere at 300 °C for 8 h (heating rate: 10 °C min^−1^) to complete the full cross-linking reaction between PAEK-COOH and PBI-4NH_2_. For PAEK-COOH, the ion-exchange capacity (IEC) value of active -COOH is 2.01 mmol g^−1^. The PBI-4NH_2_ crosslinker possesses two pair of diamino groups, it can connect the carbonxylic acid groups in different PAEK polymer chains and then result in a crosslinked structure. After crosslinking, all carboxylic acid groups reacted with amino groups to afford new imidazole groups to absorb PA molecules by acid-base interaction during PA immersing. The capacity of imidazole group in PAEK-*cr*-PBI is calculated as 2.64 mmol g^−1^.

Semi-IPN/*x*PBI membranes: PAEK-COOH and OPBI were firstly dissolved in NMP at 80 °C to form a transparent solution. Then, a solution with a specific amount of PBI-4NH_2_ in NMP was added dropwise into the above solution with a total solid content of approximately 10 wt.%, and the requisite content of PAEK-COOH, PBI-4NH_2_, and OPBI was controlled. Following this, a solution of PAEK-COOH, OPBI, and PBI-4NH_2_ in NMP was obtained for fabricating semi-IPN/*x*PBI membranes using the same preparation procedure of PAEK-*cr*-PBI membrane.

### 2.3. Characterization and Measurements

#### 2.3.1. Characterization of Chemical Structure

^1^H NMR spectra of PBI-4NH_2_ and PAEK-COOH were collected by a Bruker DRX-500 spectrometer (PerkinElmer Inc., Waltham, MA, USA) utilizing deuterated dimethyl sulfoxide (DMSO-d_6_) as the solvent and tetramethylsilane (TMS) as the internal standard.

Fourier transform infrared (FT-IR) spectroscopy of the membranes involved in our work was conducted on a Thermo Scientific Nicolet 6700 FT-IR spectrometer (Nicolet, Waltham, MA, USA) with a wavenumber range of 4000–650 cm^−1^. The molecular weight of the synthesized PAEK-COOH was measured by gel permeation chromatography (GPC) (Headwaters Incorporated, South Jordan, UT, USA) which includes a Waters 1515 iscoratic HPLC pump, a Waters 717 plus autosampler and a Waters 2417 refractive index detector. DMF was used as the solvent at a flow rate of 1 mL/min at room temperature. The concentration of PAEK-COOH dissolved in DMF was 1 mg/mL.

#### 2.3.2. Thermal Stability

Thermogravimetric analysis (TGA) curves of the membranes were performed using a Pyris 1 TGA analyzer from the Perkin Elmer company (PerkinElmer Inc., Waltham, MA, USA), and during the testing, the temperature was raised from room temperature to 600 °C at a heating rate of 10 °C min^−1^ along with the O_2_ flow of 25 mL min^−1^. Before testing, all the dry membranes were preserved in a vacuum drying chamber at 120 °C for 12 h to completely remove residual organic solvents and the chemically bound water.

#### 2.3.3. Gel Fraction Test

A gel fraction test of semi-IPN/*x*PBI membranes was carried out by solvent extraction to reflect the crosslinking degree. OPBI and PAEK-*cr*-PBI membranes were also measured for comparison. The gel content was estimated from the residual mass of the membranes after soaking them in NMP solvent at 100 °C for 48 h. Before testing, the mass of the dried membranes was denoted as *W*_1_. After the test, the membranes were washed several times using deionized water and then dried at 120 °C for 24 h in the vacuum drying oven until the mass was constant. They were then reweighed (*W*_2_). The *Gel fraction* is calculated using following equation:

(1) $$Gel\;fraction\;\left(\%\right)=W_{2}/W_{1}\times 100$$

#### 2.3.4. PA Uptake and Volumetric Swelling Ratio

The PA uptake and volumetric swelling ratio of the membranes were calculated by virtue of measuring the changes between the wet and dry weight and dimension of the membranes. Dried OPBI, PAEK-*cr*-PBI, and semi-IPN/*x*PBI membranes were used for testing. Before PA doping, all membranes were dried at 100 °C for 24 h in a vacuum drying oven to reach constant weight, and then the weight, length, width, and thickness of the acid-undoped dry membranes were recorded, respectively. After that, the dry membranes were immersed in 85 wt.% PA solution at 120 °C for 15 h. Excess PA on the surface of the membranes was immediately wiped with tissue paper as soon as they were taken out of the PA solution, and their corresponding weight and size were measured. The PA-doped membranes were dried at 100 °C for 12 h in a vacuum drying oven to get rid of the bound water, and their weight and dimension were recorded. A total of three samples were taken for each membrane to acquire the average value.

Finally, the *PA uptake* and volumetric *Swelling ratio* of the membranes was calculated according to the following equations

(2) $$PA\;uptake\;\left(\%\right)=\frac{W_{doped}-W_{undoped}}{W_{doped}}\times 100\;$$

(3) $$Swelling\;ratio\;\left(\%\right)=\frac{V_{wet}-V_{undoped}}{V_{undoped}}\times 100$$

where *W_undoped_* and *W_doped_* are the weight and volume of the dry membrane and PA-doped membrane, respectively. *V_undoped_* and *V_doped_* are assigned to the volume of the dry membrane and PA-doped membrane, respectively.

#### 2.3.5. Evaluation of Oxidative Stability

The oxidative stability of PEMs has generally been assessed by oxidative radicals to suggest the long-term durability of the membranes. OPBI, PAEK-*cr*-PBI, and semi-IPN/*x*PBI membranes were submersed in a Fenton reagent (3 wt.% H_2_O_2_ solution containing 4 ppm Fe^2+^) at 80 °C for 48 h, then the samples were collected, rinsed with deionized water, and dried in an oven at 120 °C for 24 h. To ensure the accuracy of the results, three samples were treated in parallel for each membrane, and the final results were averaged. The oxidative stability (*OS*, %) was calculated by the retained weight after immersion using Equation (4):

(4) $$OS\;\left(\%\right)=W_{4}/W_{3}\times 100$$

where *W*_3_ is the weight of the sample before the test and *W*_4_ is the residue weight of the membrane after the test.

#### 2.3.6. Mechanical Properties

The mechanical properties of PA doped OPBI, PAEK-*cr*-PBI, and semi-IPN/xPBI membranes were investigated by a universal testing instrument (New SANS, Shenzhen, China), and the membrane samples (40–50 mm length and 4–6 mm width) were conducted at room temperature at a constant strain rate of 5 mm min^−1^. Prior to testing, the samples were dried at 100 °C for 24 h. For each data item obtained, at least three samples were tested to obtain an average value.

#### 2.3.7. Proton Conductivity

The proton conductivities of PA doped membranes were measured via a four-electrode AC impedance method employing a PGSTAT204 electrochemical workstation (AUT50992, AUTOLAB) with a frequency range of 0.1 Hz to 1 × 10^5^ Hz. The impedance measurement of rectangular membrane samples was performed in the absence of humidification with a temperature range of 80~180 °C. The conductivity σ (S cm^−1^) was calculated from the following equation:

(5) $$\sigma =\frac{L}{R\times A}$$

where *L* (cm), *A* (cm^2^), and *R* (Ω) are the distance between two electrodes, the cross-sectional area of the membranes, and the membrane resistance derived from the Nyquist plot, respectively.

#### 2.3.8. Single Cell Test

The PA doped membranes were applied to fabricate membrane electrode assemblies (MEAs) with an active area of 5 cm^2^. The 1.0 mg cm^−2^ Pt/C electrodes were purchased from Shanghai Hensen Company (Shanghai, China). The single cell performance was operated on a fuel cell testing system for dry H_2_ and O_2_ before and after optimizing test conditions.

## 3. Results and Discussion

### 3.1. Chemical Structure Identification

With regard to the characterization of molecular weight correlation, the GPC results show the number-average molar mass, Mn = 159,500 g mol^−1^, the weight-average molar mass, Mw = 162,600 g mol^−1^, and the polydispersity, PDI = 1.02, suggesting a high molecular weight acquired, which is the prerequisite for the formation of a membrane.

**Figure 1:**
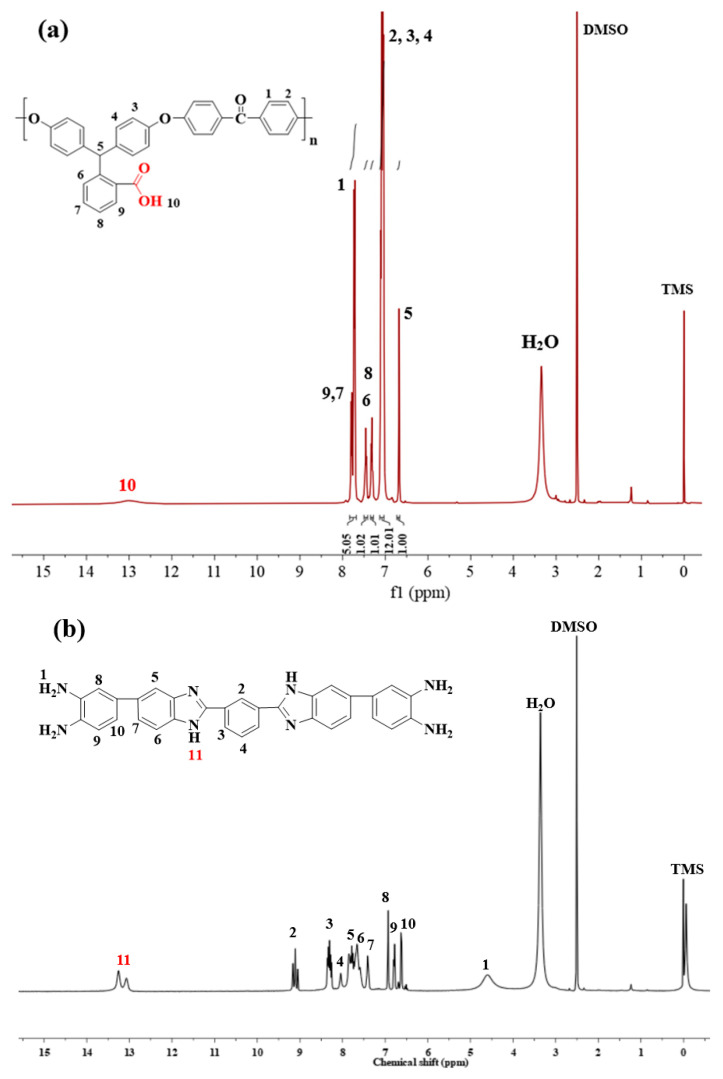
^1^H NMR spectra of (**a**) PAEK-COOH, and (**b**) PBI-4NH_2_ in DMSO-d_6_.

The unambiguous structure of PAEK-COOH and PBI-4NH_2_ was identified by a ^1^H NMR (500 MHz, DMSO-d_6_) spectrum, as shown in Figure 1 For PAEK-COOH (Figure 1a). The chemical shift at 6.68–7.80 ppm is attributed to the aromatic protons, and the peak at about 13.05 ppm is assigned to the proton in the carboxylic acid group. Similarly, for PBI-4NH_2_ (Figure 1b), the associated signal peaks are assigned from number 1 to 11. The two peaks at 13.07 and 13.25 ppm are designated as the imidazole proton. The signal peaks appearing in between 6.63 and 6.93 ppm are attributed to the terminal aromatic proton. Furthermore, the peaks in the range of 8.05–9.16 ppm correspond to protons of the benzene ring between two benzimidazole rings. At the same time, the benzene ring protons adjacent to the imidazole ring are at between 7.41 and 7.83 ppm, and the proton peak of terminal amine emerged at 4.61 ppm. The above analysis indicates that the structure of PAEK-COOH and PBI-4NH_2_ are in good agreement with the spectral data.

**Figure 2:**
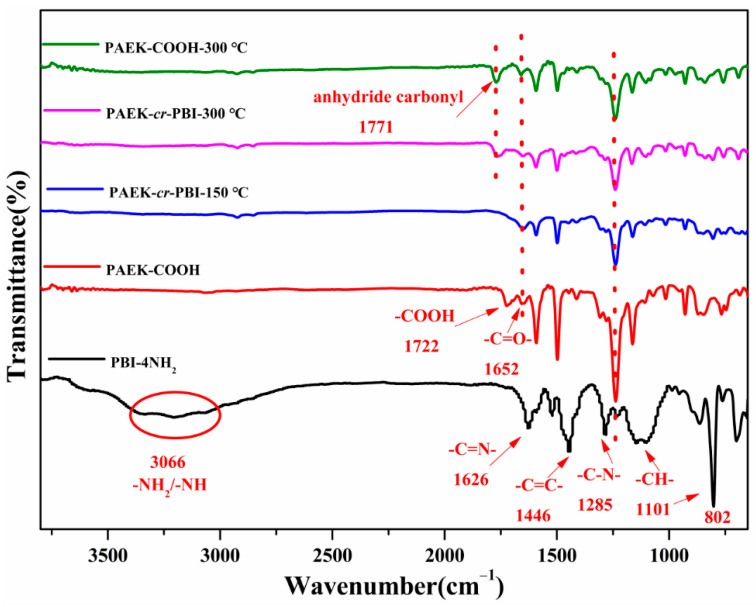
FT-IR spectra of PAEK-COOH, PBI-4NH_2_, and PAEK-*cr*-PBI with different treatment temperatures.

The structure changes of PAEK-COOH and PBI-4NH_2_ before and after crosslinking were also verified by FT-IR to speculate the possible mechanism of the reaction, as presented in Figure 2.

**Scheme 3:**
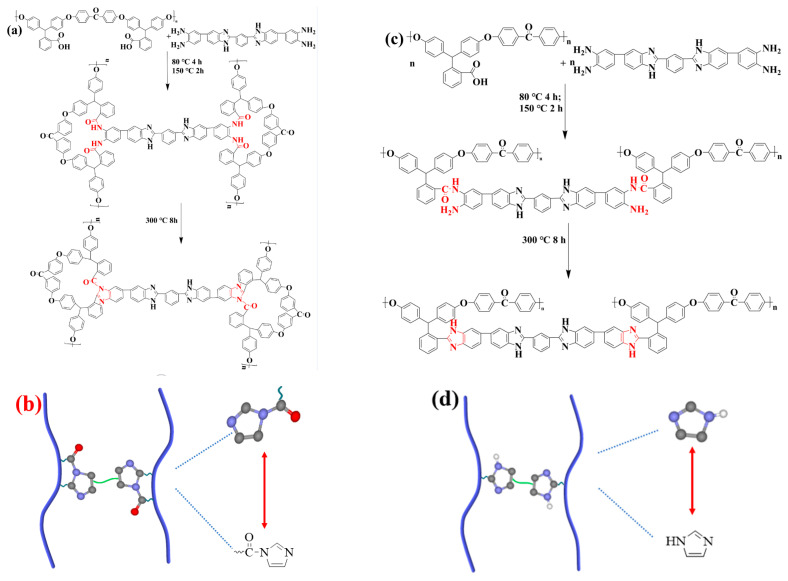
Cross-linking mechanism and corresponding structure diagram of PAEK-*cr*-PBI membrane to form acyl benzimidazole (**a**,**b**) and benzimidazole (**c**,**d**).

For PBI-4NH_2_, the typical characteristic absorption peaks at 3066 cm^−1^, 1626 cm^−1^, 1446 cm^−1^, and 1285 cm^−1^ are ascribed to the stretching vibration of the -NH_2_ or -NH group, the in-plane C=C deformation of benzimidazole rings, and the C=N and C-N stretching vibrations of imidazole, respectively. Additionally, the absorption bands at approximately 1101 cm^−1^ and 802 cm^−1^ imply the existence of the benzene ring. These features indicate that the PBI-4NH_2_ has been successfully prepared. For PAEK-COOH, the main vibration peaks of the carbonyl (C=O) stretching of the carboxylic acid group (1722 cm^−1^) and ketone (1652 cm^−1^) can be obviously observed. Meanwhile, a significant absorption band centered at 1161 cm^−1^ matches with the Ar-O-Ar linkage, manifesting the successful synthesis of PAEK-COOH. After heat treatment at 300 °C (PAEK-COOH-300 °C), the absorption peak of the carboxylic acid groups at 1722 cm^−1^ disappears and a new vibration peak appears at 1771 cm^−1^, which may be due to the dehydration between the carboxylic acid groups to form anhydride carbonyl. Similarly, for the pre-crosslinked membrane (PAEK-*cr*-PBI-150 °C), the disappearance of the -COOH absorption peak is due to the reaction of -COOH and -NH_2_ to yield the amide group. Thereafter, a new vibration peak at 1765 cm^−1^ may be attributed to the formation of acyl imidazole. In addition, it is worth noting that the vibration bands of C=N or C–N are superimposed by other function groups in the backbone of the crosslinked membrane and they are not recognized from PAEK-COOH. The disappearance of the strong absorption band of -COOH also hints that -COOH groups are converted into benzimidazole groups. Therefore, it can be inferred from the above discussion that two reactions occur during the crosslinking process, and the possible reaction scheme is depicted in Scheme 3. A model reaction (Figure S1 and Scheme S1 in Supplementary Materials) was designed to further confirm the possible reactions, and the results are consistent with the above hypotheses.

**Figure 3:**
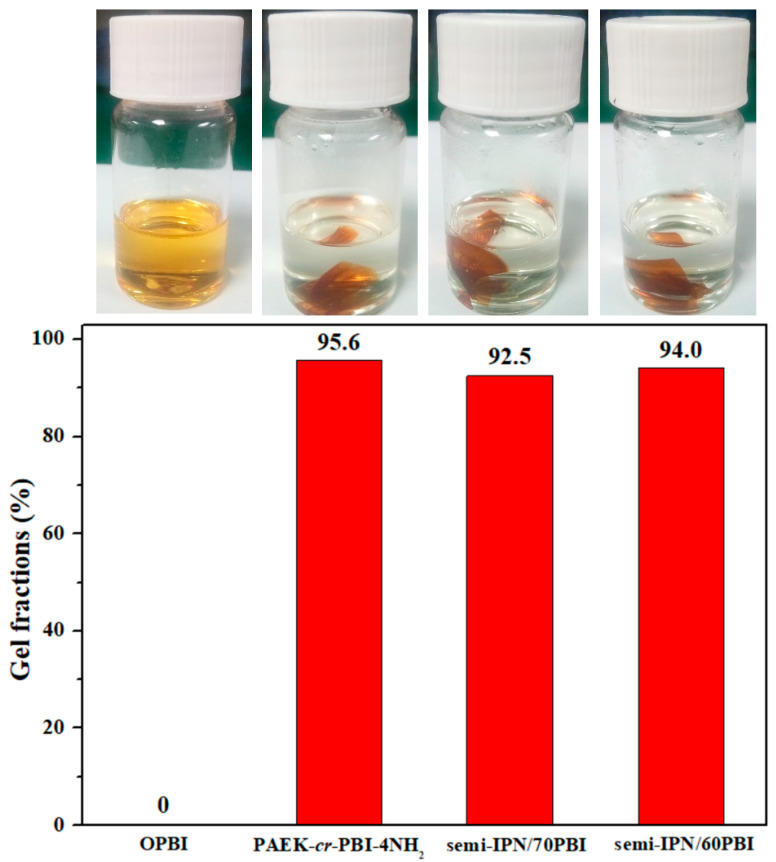
Gel fractions of OPBI, PAEK-*cr*-PBI, and semi-IPN/*x*PBI membranes after immersing in NMP at 100 °C for 48 h.

The crosslinked network will result in the insolubility of membranes in common organic solvent. The gel fractions test was performed to evaluate the cross-linking degree of PAEK-*cr*-PBI and semi-IPN/*x*PBI, and the results are shown in Figure 3. After soaking in NMP for 48 h at 100 °C, it can be clearly observed that semi-IPN/*x*PBI membranes keep almost wholly intact, while the pure OPBI membrane is entirely dissolved. The gel fractions of PAEK-*cr*-PBI, semi-IPN/70PBI, and semi-IPN/60PBI membranes were 95.6%, 92.5%, and 94.0%, respectively. Moreover, with the increase of OPBI content, the residual weight did not decrease significantly, indicating that the construction of a 3D network structure has a certain inhibitory effect on the molecular chain movement of OPBI.

### 3.2. Thermal and Oxidative Stability

**Figure 4:**
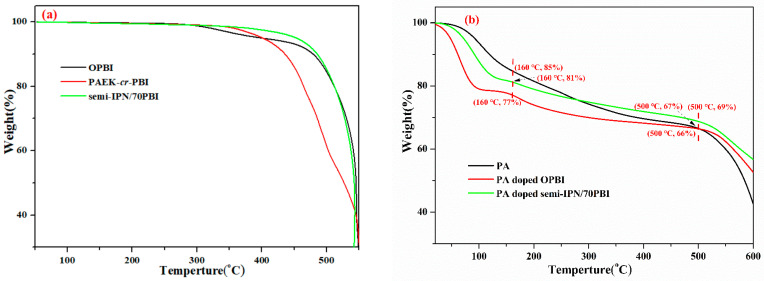
TGA curves of (**a**) PA undoped, and (**b**) PA doped OPBI, PAEK-*cr*-PBI, and semi-IPN/70PBI membranes under O_2_ flow at a heating rate of 10 °C min^−1^.

The thermal stability of the membranes before and after PA doping was evaluated using TGA under O_2_ atmosphere. As shown in Figure 4a, the thermal decomposition temperatures of PAEK-*cr*-PBI and semi-IPN/70PBI are higher than those of OPBI, implying that the crosslinked structure induced by crosslinker PBI-4NH_2_ enhances their thermal stability. Additionally, TGA profiles of PA, PA doped OPBI, and semi-IPN/70PBI membranes under O_2_ atmosphere are also presented in Figure 4b. After doping PA, both membranes lost weight at about 160 °C due to PA dehydration. For PA, the weight decreases from 85% to 67% from 160 °C to 500 °C, resulting in a loss of 18% total weight. For PA doped OPBI membrane, the weight decreases from 77% to 66% from 160 °C to 500 °C, resulting in a loss of 11% total weight. According to the theoretical calculation, the weight loss due to PA dehydration is up to 13%. For PA doped semi-IPN/70PBI composite membrane, the weight decreases from 81% to 69% from 160 °C to 500 °C, resulting in a loss of 12% total weight. According to the theoretical calculation, the weight loss due to PA dehydration is up to 12%. The theoretical value and the experimental value are within the allowable range of error, indicating that the degradation of the polymer membrane itself can be observed over a temperature range similar to that of the PA undoped polymer membrane. Meanwhile, the 5% weight loss temperatures (T_–5%_) for dry membranes under O_2_ atmosphere determined by TGA are listed in Table 1. The T_–5%_ is all above 300 °C, indicating that the thermal stability of each sample can guarantee their application in HT-PEMs.

**Figure 5:**
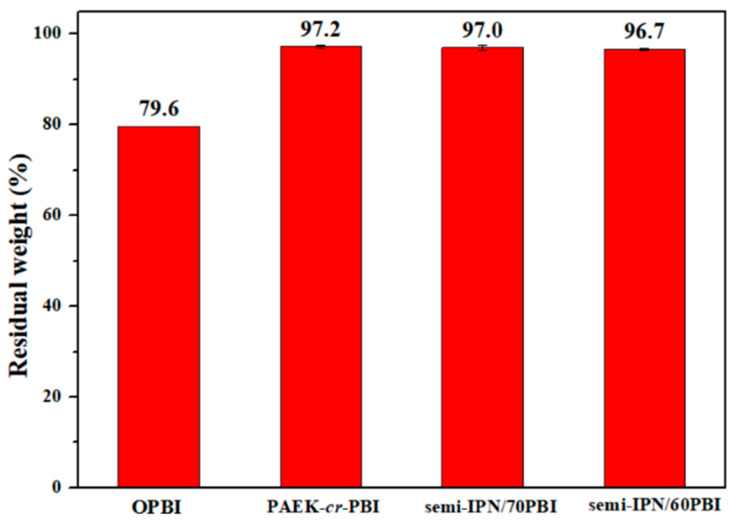
Residual weight of the membranes after treatment with Fenton reagent for 48 h at 80 °C.

Oxidative stability is a crucial factor which affects the lifetime of HT-PEMs. Figure 5 reveals the residual weight of the membranes after immersing them in Fenton reagent for 48 h at 100 °C. The pure linear OPBI membrane presents the maximum weight loss of about 20 wt.%. Meanwhile, all crosslinked composite membranes display better oxidative stability than the OPBI membrane, with a small weight loss close to 3 wt.%. The oxidation stability test results of the dry membranes showed excellent resistance to oxidation via radicals. However, given that these membranes are not swelling in water at all, all the degradation is taking part only on the surface, but not in the bulk of the membrane. In order to be closer to the conditions of reliable fuel cells, we tested the oxidation stabilities of the PA doped membranes. In the experiment, we set a gradient every 0.5 h to carry out the experiment. According to the observation, the PA doped OPBI membranes began to break after soaking in Fenton reagent for 2 h at 80 °C, and the PA doped semi-IPN/70PBI composite membranes began to break at 3.5 h. We repeatedly washed the membranes soaked for 1.5 h with deionized water to wash away PA and dried them in a vacuum oven at 100 °C. Compared with the weight of the dry membranes before PA adsorption, it is found that there was almost no weight loss, which indicates to some extent that the larger the adsorption amount of PA is, the more serious the swelling is, and the more unstable it is in Fenton reagent. Compared with PA doped OPBI membrane, PA doped semi-IPN/70PBI composite has a better antioxidant stability, which is believed to be attributed to its crosslinked 3D network structure.

### 3.3. PA Doping Level, Volumetric Swelling Ratio, and Mechanical Properties

For PA-doped HT-PEMs, ADL is the most important factor because proton transport is completed through the dissociation of PA molecules. Table 2 summarizes the ADLs and swelling ratios of PA doped membranes. It is apparent that the ADLs of PAEK-*cr*-PBI membranes are lower than those of OPBI membranes under the same PA doping condition due to the lower content of active imidazole groups. After PA doping, OPBI membrane exhibits a high volumetric swelling ratio of 140% with an ADL of 220%. The high swelling ratio of HT-PEMs deteriorates the stability of MEA. By confining OPBI in the crosslinked network, the volumetric swelling ratios of semi-IPN/*x*PBI membranes are reduced to only 60% of that of OPBI and they still have comparative ADLs, which are necessary for the proton transportation.

**Figure 6:**
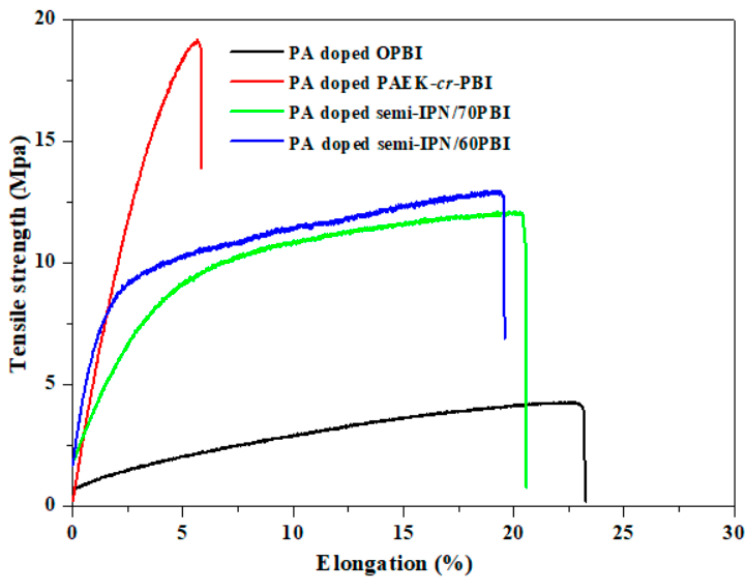
Stress-strain curves of PA doped membranes.

It is also vital for HT-PEMs to possess the required mechanical performance for use in HT-PEMFCs. The typical stress-strain curves of different membranes are depicted in Figure 6 and the detail data are displayed in Table 2. In general, in the case of crosslinked membranes, 3D networks can improve the interaction between chains, thus enhancing the mechanical properties of the membrane, and the test results are as expected. Due to the low PA adsorption, the PAEK-*cr*-PBI membrane exhibits a tensile strength of 19.2 MPa and a lower elongation at fracture value of 5.7%. For the linear OPBI membrane, the tensile strength is 5.1 MPa which is ascribed to the plasticizing effect of the large amount of doped PA. Moreover, under the effect of the crosslinking structure, two PA doped semi-IPN/*x*PBI membranes reveal higher tensile strength, along with the values of 11.8 MPa and 12.8 MPa, respectively, which are about 2.5 times higher than that of OPBI.

### 3.4. Proton Conductivity 

**Figure 7:**
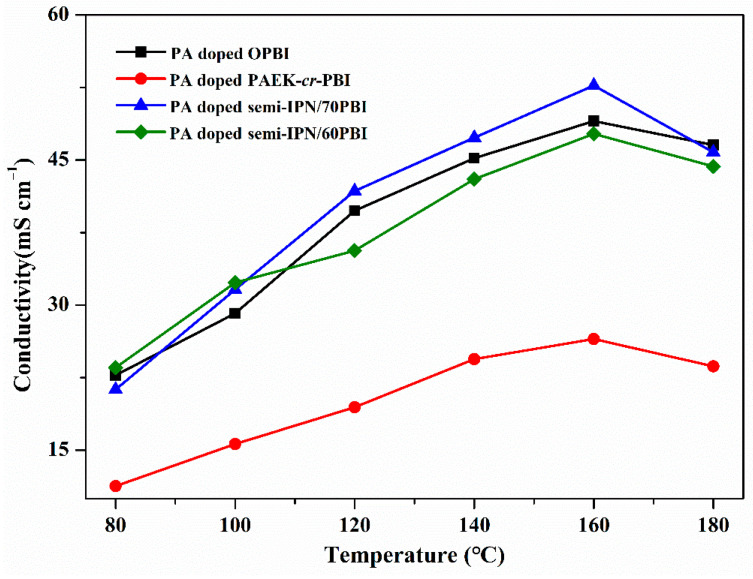
Proton conductivities of PA doped membranes.

The proton conductivities of the membranes were measured from 80 °C to 180 °C under anhydrous condition. As shown in Figure 7, the proton conductivities of all membranes increase with increasing temperature, and a slight decrease occurs above 160 °C which is ascribed to the dimerization of PA at this temperature [45]. The PA doped PAEK-*cr*-PBI membrane has the lowest conductivity of 27 mS cm^−1^ at 160 °C as it has the lowest active group content. The proton conductivities of PA doped semi-IPN/*x*PBI are almost equivalent to those of the PA doped OPBI membrane, even though its ADL is lower than that of the OPBI membrane. The PA doped semi-IPN/70PBI possesses a proton conductivity of 53 mS cm^−1^ at 160 °C, which is higher than that of the PA doped OPBI membrane (49 mS cm^−1^), indicating that the construction of a 3D crosslinked network with active imidazole groups is conducive to the formation of a well-connected proton conductivity channel.

### 3.5. Single Fuel Cell Performance

**Figure 8:**
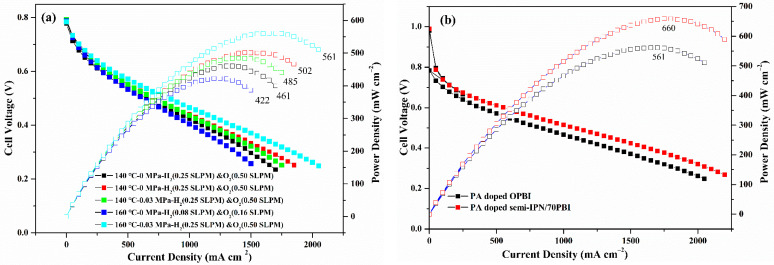
Polarization curves (solid symbols) and power densities (open symbols) of single fuel cells based on the (**a**) PA doped OPBI under different test conditions, and (**b**) PA doped OPBI and semi-IPN/70PBI membranes with flow rates of 250 and 500 mL min^−1^ for dry H_2_ and O_2_ at a backpressure of 0.03 MPa. The thickness of PA doped OPBI and PA doped semi-IPN/70PBI is 65 and 48 μm, respectively.

The PA doped semi-IPN/70PBI membrane with a high proton conductivity and excellent mechanical strength was selected to evaluate single fuel cell performance. Meanwhile, the PA doped OPBI membrane was also employed as a control. The effects of temperature, back pressure, and gas flow rate (SLPM) on single cell performance with the PA doped OPBI membrane were investigated, and the cell performances under different test conditions are shown in Figure 8a. The best performance of a single cell was obtained at 160 °C with a background pressure and high flow rate of H_2_ and O_2_. Figure 8b reveals the polarization and power density curves of the single HT-PEMFCs with the PA doped OPBI and semi-IPN/70PBI membranes under the optimized condition. Compared to the pristine OPBI, the MEA with semi-IPN/70PBI displays a higher peak power density. The open-circuit voltages (OCVs) of MEAs with semi-IPN/70PBI and OPBI membranes are 0.982 and 0.788 V, respectively, implying that semi-IPN/70PBI membranes possess a low gas permeability. At 160 °C, along with flow rates of 250 and 500 mL min^−1^ for dry H_2_ and O_2_ at a backpressure of 0.03 MPa, semi-IPN/70PBI gives a maximum peak power density of 660 mW cm^−2^, which is notably 18% higher than that of the pristine OPBI membrane (561 mW cm^−2^) with almost the same ADLs. This result demonstrates that constructing a dense cross-linked 3D network substrate with active imidazole groups is a potential methodology to enhance the performance of HT-PEMFCs.

## 4. Conclusions

In this work, it was shown that a series of semi-IPN/70PBI membranes with a semi-interpenetration network structure can be readily prepared using a solution casting method via succeeding stepwise heating. Due to the construction of a 3D crosslinked network, the properties of semi-IPN/*x*PBI such as oxidative stability, volumetric swelling ratio, mechanical properties, and proton conductivity were greatly enhanced compared with the pristine linear OPBI membrane. The PA doped semi-IPN/70PBI membrane shows a 2.5 times higher tensile strength (11.8 MPa) and higher proton conductivity at 160 °C (53 mS cm^−1^) compared to the PA doped OPBI membrane. In particular, a single H_2_/O_2_ HT-PEMFC assembled with a semi-IPN/70PBI membrane reaches a maximum power density of 660 mW cm^−2^ at 160 °C with flow rates of 250 and 500 mL min^−1^ for dry H_2_ and O_2_ at a backpressure of 0.03 MPa, which is 18% higher than that of the pristine OPBI membrane (561 mW cm^−2^) under the same test condition. In conclusion, this work demonstrates a feasible and effective method to construct a composite membrane with a 3D semi-interpenetration network structure bearing abundant active imidazole groups for potential and extensive application in HT-PEMFCs.

## Figures and Tables

**Table 1:** 5% weight loss temperature for dry membrane.

Sample	OPBI	PAEK-*cr*-PBI	Semi-IPN/70PBI
T_–5%/_°C	399	401	453

**Table 2:** PA uptake, swelling, and mechanical properties of all PA doped membranes.

Membrane	PA Uptake (%)	S_volume_ (%)	Tensile Strength (MPa)	Elongation (%)
OPBI	219 ± 3	128 ± 12	5.1 ± 0.8	22.1 ± 2.2
PAEK-*cr*-PBI	121 ± 2	46 ± 3	20.4 ± 1.2	4.6 ± 1.1
Semi-IPN/70PBI	191 ± 1	78 ± 3	11.8 ± 0.3	20.3 ± 4.8
Semi-IPN/60PBI	179 ± 5	72 ± 3	12.8 ± 0.2	18.6 ± 1.9

## Data Availability

Not applicable.

## Supplementary Materials

The following supporting information can be downloaded at: https://www.mdpi.com/article/10.3390/nano12050773/s1, Figure S1: FT-IR spectroscopy of model compound; Scheme S1: Possible reaction mechanism of model compound.
