# Supplementary material for: Polybenzimidazole Confined in Semi-Interpenetrating Networks of Crosslinked Poly (Arylene Ether Ketone) for High Temperature Proton Exchange Membrane
Source: Nanomaterials (Basel). 2022 Feb 25;12(5):773. doi: 10.3390/nano12050773 (PMC8912004; doi:10.3390/nano12050773)
Supplement: Supplementary file 1 [file nanomaterials-12-00773-s001.zip › nanomaterials-1559732-supplementary.pdf]

nanomaterials

Supplementary Materials

Polybenzimidazole Confined in Semi-Interpenetrating Networks of Crosslinked Poly (arylene ether ketone) for High Temperature Proton Exchange Membrane

Erli Qu [1], Junqiao Jiang [1], Min Xiao [1], Dongmei Han [1,2], Sheng Huang [1], Zhiheng Huang [1], Shuanjin Wang [1,*] and Yuezhong Meng [1,*]

1. The Key Laboratory of Low-carbon Chemistry & Energy Conservation of Guangdong Province, State Key Laboratory of Optoelectronic Materials and Technologies, School of Materials Science and Engineering, Sun Yat-sen University, Guangzhou 510275, China; querli@mail2.sysu.edu.cn (E.Q.); jiangjq5@mail2.sysu.edu.cn (J.J.); stsxm@mail.sysu.edu.cn (M.X.); handongm@mail.sysu.edu.cn (D.H.)huangsh47@mail.sysu.edu.cn (S.H.); hzh29@mail.sysu.edu.cn (Z.H.)
2. School of Chemical Engineering and Technology, Sun Yat-Sen University, Zhuhai 519082, China

* Correspondence: wangshj@mail.sysu.edu.cn (S.W.); mengyzh@mail.sysu.edu.cn (Y.M.)

Citation: Qu, E.; Jiang, J.; Xiao, M.; Han, D.; Huang, S.; Huang, Z.; Wang, S. Polybenzimidazole Confined in Semi-Interpenetrating Networks of Crosslinked Poly (arylene ether ketone) for High Temperature Proton Exchange Membrane. *Nanomaterials* **2022**, *12*, 773. https://doi.org/10.3390/nano12050773

Academic Editors: Ioannis V. Yentekakis; David Marrero-López

Received: 31 December 2021
Accepted: 23 February 2022
Published: 25 February 2022

Publisher's Note: MDPI stays neutral with regard to jurisdictional claims in published maps and institutional affiliations.

Copyright: © 2022 by the authors. Submitted for possible open access publication under the terms and conditions of the Creative Commons Attribution (CC BY) license (https://creativecommons.org/licenses/by/4.0/).

Model reaction

In order to figure out the structure of the crosslinked resultant, a model reaction was performed a condensation reaction between 4-Methoxy-o-phenylenediamine (4M-OPD) and poly (arylene ether ketone) with the pendant carboxyl group (PAEK-COOH) as described below. The chemical structure of the reaction product (PAEK-4M-OPD) was confirmed by FTIR in Figure S1.

The model reaction was performed via a two-stage process as follows: firstly, the mixed solution of 0.1990 g (0.4 mmol -COOH groups) PAEK-COOH and 0.0556 g (0.4 mmol) 4M-OPD were dissolved 4.5 mL NMP to form a solution and then heated at 80 °C for 4 h, 150 °C for 2 h to form carboxamide (O=C-N-) bonds between the carboxylic acid groups (-COOH) of PAEK-COOH and amino groups of 4M-OPD, and then, at 300 °C for 8 h to create the C=N bond. Additionally, in order to complete the reaction of -COOH, the experiment with the molar ratio of 1:2 (-COOH to 4M-OPD) was carried out as a comparison.

The results of FT-IR spectra are shown in Figure S1. It is worth noting that the C=N or C-N vibration peak was overlay by other function groups in the main-chain of the model product (PAEK-OPD) and not distinguished from PAEK-COOH.

However, it can be seen from Figure S1 that the strong vibration peak at1722 cm^{-1} owning to the -C=O asymmetric stretching of -COOH in PAEK-COOH was weakened or even disappeared in the pre-reaction stage at 150 °C, and the vibration peak of -C=O (1653 cm^{-1}) becomes wider, which is the result of the comprehensive action of -C=O in -CO-NH-group (1680-1630 cm^{-1}) and -C=N- (1660-1630 cm^{-1}) in imidazole ring. The formation of imidazole ring in the pre-reaction stage can be preliminarily judged. In addition, compared to pre-reacted resultant, after 300 °C heat treatment, a new vibration peak appeared at 1769 cm^{-1}, which is attributed to the conversion of intermediate amide to acyl imidazole. Finally, that is, implying a comprehensive conversion of almost all of the -COOH into benzimidazole groups or acyl benzimidazole groups.

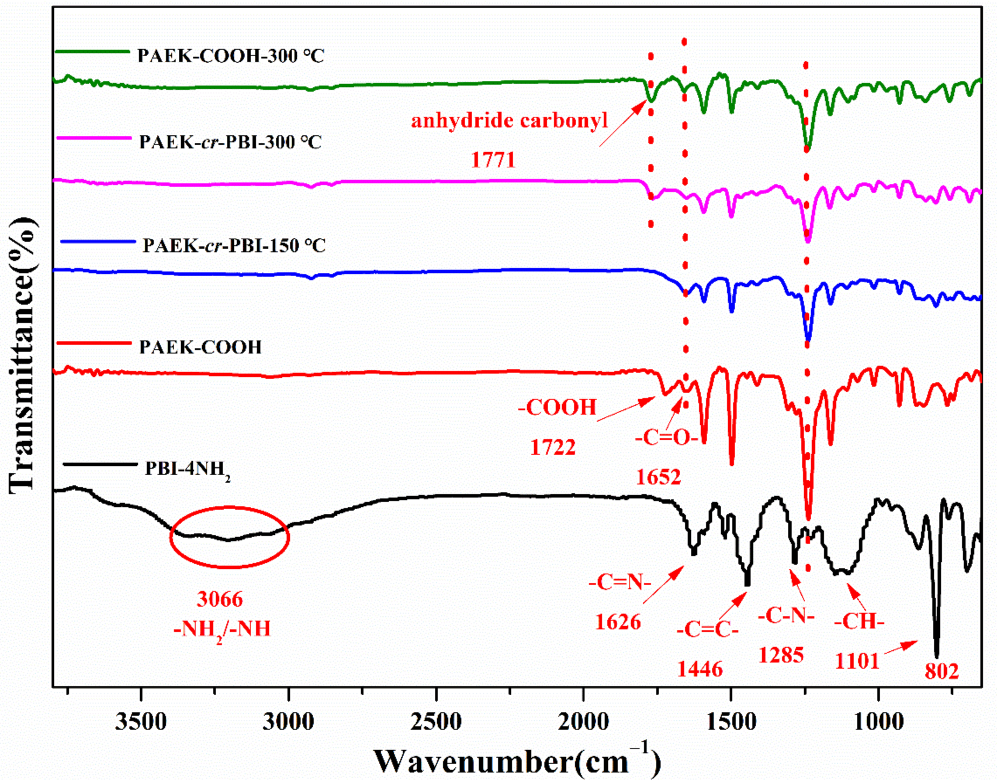

Figure S1. FT-IR spectroscopy of model compound.

According to the above analysis, there are two kinds of reaction mechanism for the model reaction. The product formation process is shown **in Scheme S1.**

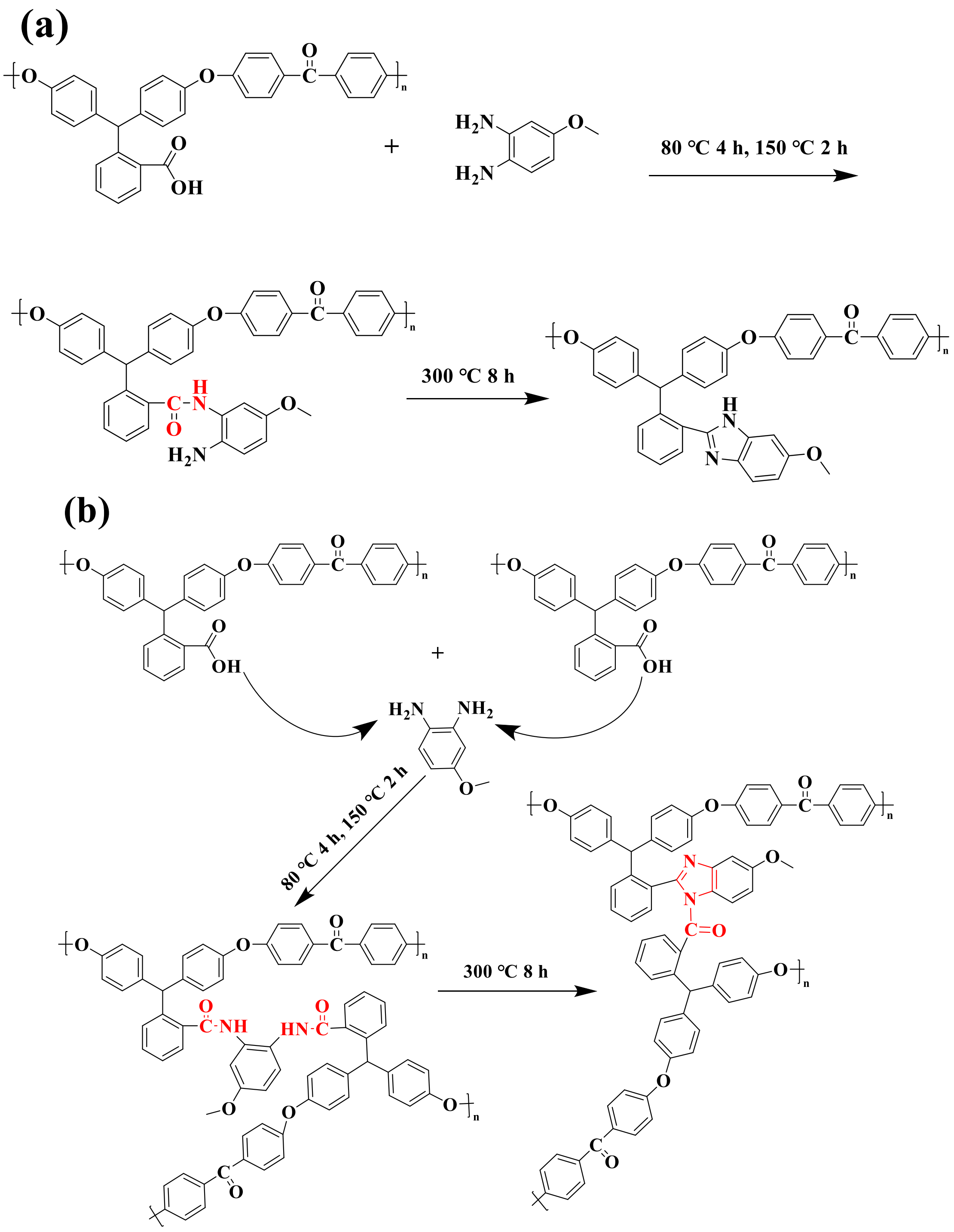

Scheme S1. Possible reaction mechanism of model compound to form benzimidazole (**a**) and acyl benzimidazole (**b**).
